# Paraproteinemic neuropathy: a practical review

**DOI:** 10.1186/s12883-016-0532-4

**Published:** 2016-01-28

**Authors:** Richard A. Rison, Said R. Beydoun

**Affiliations:** University of Southern California, Keck School of Medicine, Los Angeles County Medical Center, Medical Director PIH Health-Whittier Stroke Program, Neurology Consultants Medical Group, 12401 Washington Blvd., Whittier, CA 90602 USA; University of Southern California, Keck School of Medicine, Los Angeles County Medical Center, 1520 San Pablo Street Suite 3000, Los Angeles, CA 90033 USA

**Keywords:** Paraproteinemia, Neuropathy, Monoclonal gammopathy, Electrodiagnostic studies, Intravenous immunoglobulins, Hematologic malignancy, Diagnosis, Treatment

## Abstract

The term paraproteinemic neuropathy describes a heterogeneous set of neuropathies characterized by the presence of homogeneous immunoglobulin in the serum. An abnormal clonal proliferation of B-lymphocytes or plasma cells, which may or may not occur in the context of a hematologic malignancy, produces the immunoglobulins in excess. If malignancy is identified, treatment should be targeted to the neoplasm. Most cases, however, occur as monoclonal gammopathy of undetermined significance. Few prospective, randomized, placebo-controlled trials are available to inform the management of paraproteinemic neuropathies. Clinical experience combined with data from smaller, uncontrolled studies provide a basis for recommendations, which depend on the specific clinical setting in which the paraprotein occurs. In this review, we provide a clinically practical approach to diagnosis and management of such patients.

## Introduction

Peripheral neuropathy is defined as a disease or degenerative state of the peripheral nerves in which motor, sensory, or vasomotor nerve fibers are affected. The condition appears clinically as muscle weakness and atrophy, pain, and numbness [1]. Several monoclonal antibody-producing conditions are associated with peripheral neuropathy, and in these circumstances, the constellation of neurological symptoms are often referred to as paraproteinemic neuropathy (PPN) [2, 3]. As neuropathy is relatively common with M-protein and vice-versa, PPN may therefore be defined further as a heterogeneous group of neuropathies, which share the common feature of a homogeneous immunoglobulin in the serum [4]. Typically manifested neurologically as a length-dependent axonal loss sensorimotor polyneuropathy, PPN affects some or all sensory modalities causing allodynia, hyperpathia, cramps, or mild distal weakness (rarely it can be associated with more profound motor symptoms). Sometimes there is multiorgan involvement. Peripheral neuropathy symptoms may precede by years other clinical symptoms or diagnosis of the antibody-producing condition, whether it be hematologic malignancy or monoclonal gammopathy of undetermined significance [5]. Therapies depend on the particular PPN subtype and the pathophysiology involved, and range from intravenous immunoglobulin (IVIG), plasma exchange, and corticosteroids to rituximab and various chemotherapies.

## Background

### Paraproteinemia

Paraproteins are immunoglobulins that are produced in excess by an abnormal clonal proliferation of B-lymphocytes or plasma cells. These monoclonal proteins exist as heavy chain subtypes (IgG, IgA, IgG, and less commonly IgD or IgE) and light chain subtypes (kappa or lambda) [1]. Clonal proliferation may occur in the context of a hematologic malignancy or a premalignancy. Commonly associated disorders include multiple myeloma, cryoglobulinemia, lymphoma, amyloidosis, Waldenstrom macroglobulinemia, and POEMS (polyneuropathy, organomegaly, endocrinopathy, M-protein spike, and skin manifestations) syndrome [6].

Despite the range of associated hematologic disorders, paraproteins most commonly occur as a monoclonal gammopathy of undetermined significance (MGUS) [7, 8]. MGUS is a common, age-related medical condition characterized by an accumulation of bone marrow plasma cells derived from a single abnormal clone without proliferation of malignant cells [8–10]. Three criteria define MGUS: A monoclonal paraprotein band less than 30 g/L (3 g/dL); plasma cells less than 10 % on bone marrow examination; and no evidence of bone lesions, anemia, hypercalcemia, or renal insufficiency related to the paraprotein [11]. MGUS-associated neuropathies are generally not treated, except in the case of a disabling IgM monoclonal gammopathy or when associated with chronic inflammatory demyelinating neuropathy (CIDP), as in certain cases of IgG or IgA monoclonal gammopathy. CIDP-MGUS (non-IgM) has the same clinical and electrodiagnostic characteristics as pure CIDP and has the same treatment choice and algorithms (please note however that many neurologists still try standard CIDP treatments first before considering immunosuppression, even though they are not as effective as in idiopathic CIDP) [12]. In the case of IgG monoclonal gammopathy, studies have shown improvement on impairment measures following treatment with rituximab, cyclophosphamide/prednisone, or fludarabine [11–13].

Multiple myeloma is part of the spectrum of diseases ranging from MGUS to plasma cell leukemia, and similar to MGUS, is characterized by proliferation of a plasma cell clone and subsequent overabundance of monoclonal paraprotein (M protein) [13].

### Paraproteinemic neuropathy (PPN)

Several disorders of the peripheral nervous system are closely associated with the presence of excessive amounts of abnormal immunoglobulins in the blood [14]. PPN may be caused by interaction of antibodies with specific antigenic targets on peripheral nerves or by deposition of immunoglobulins or amyloid. The clinical presentation, treatment, and prognosis of PPN differs based on the subtype and associated disorders [3]. There are three major clinical PPN subtypes:

- Distal demyelinating symmetric neuropathy
- Chronic inflammatory demyelinating polyneuropathy (CIDP) –like
- Axonal sensorimotor peripheral neuropathy

Approximately 10 % of patients with a chronic sensorimotor neuropathy of unknown origin have an associated serum monoclonal gammopathy, and two-thirds of such cases are initially classified as MGUS [15, 16].

Clinical presentation of peripheral neuropathy associated with monoclonal proteins includes general symptoms of foot numbness, paresthesias, imbalance, gait ataxia, dysesthesia, and lancinating pain. In early stages, general signs include abnormal sensation in the legs referable to conduction in large fibers (touch, joint position, vibration). As the illness progresses, weakness in distal muscles with variable atrophy occurs [15, 17].

Paraproteins are detected in the serum of approximately 1 % of the general population [1, 7]. The prevalence of paraproteinemia rises with age, up to 5.3 % among individuals over 70 years and up to 10 % in people older than 80 years [10]. Among patients with cryptogenic neuropathy, the prevalence of paraproteinemia is 10 %. PPN is most commonly observed with IgM gammopathy (48 %), followed by IgG (37 %), and IgA (15 %). African Americans have a higher prevalence of monoclonal gammopathy [5].

## Paraproteinemic disorders

Each of the individual paraproteinemic disorders exhibits a distinct neuropathic phenotype, and the typical clinical features are described below. The prognosis is often not well-defined and unknown in many cases.

Anti-MAG neuropathy is described as “distal acquired demyelinating symmetric” (DADS) sensory and motor neuropathy. It is usually very slowly progressive and predominately distal with sensory ataxia, little or no weakness, and frequent tremor. This condition usually has a benign course with little functional deterioration over time; however, the neuropathy may evolve more rapidly at certain stages. At least one report showed that 10 and 15 years after onset, 24 % and 50 % of patients were disabled, respectively [18].

IgG/A MGUS with associated neuropathy resembling chronic inflammatory demyelinating polyneuropathy (CIDP) is a relapsing or progressive sensori-motor disorder involving the peripheral nerves with symmetrical proximal and distal weakness of the four limbs, sensory involvement and areflexia. About 80 % of patients respond to one of the typical CIDP treatments (please see the treatment section). Some patients stabilize without therapy [19].

IgG/A MGUS with associated axonal neuropathy is a sensory or sensori-motor axonal neuropathy involving distal extremities in a length-dependent fashion. Initial presentation typically entails distal lower limb sensory symptoms and signs, with motor weakness at later stages. The progression is slow and often does not require any treatment [17].

Cryoglobulinemia (most frequently mixed cryoglobulinemia) with neuropathy is characterized by multifocal axonal neuropathy, as a mononeuropathy multiplex pattern, secondary to necrotizing vasculitis. Pain can be a distinguishing feature of cryoglobulenic neuropathy. Sensory fibers are more commonly affected than motor fibers, with approximately 5 % of patients experiencing pure motor neuropathy [20–22].

Primary (AL) amyloidosis coexists with multiple myeloma in 10 % of cases, and 20 % of patients with AL present with a neuropathy [14]. The neuropathy itself is mostly symptomatic in the distal lower limbs, predominately sensory, and of the small fiber painful type. Autonomic dysfunction is frequent. Symptoms of amyloidosis include pain, weight loss, macroglossia, organomegaly, or cardiomyopathy. If left untreated, it has a poor prognosis with median survival less than 18 months from onset [23–25].

CANOMAD syndrome (Chronic Ataxic Neuropathy with Ophthalmoplegia, M-protein, cold Agglutinins and Disialosyl antibodies [anti-ganglioside, anti-GD1b, and anti-GQ1b]) is a rare phenotype associated with an IgM MGUS [26]. It may correspond to a chronic form of Miller Fisher syndrome, a rare variant of Guillain–Barré syndrome that manifests with ataxia, areflexia, and ophthalmoplegia. Ataxia is profound, severely impairing function, but motor strength remains relatively spared [27].

POEMS (Polyneuropathy, Organomegaly, Endocrinopathy, M-protein, Skin changes) syndrome is a rare entity featuring sclerotic bone lesions, Castleman’s disease (a very rare disorder characterized by non-cancerous growths that may develop in the lymph node tissue at a single site or throughout the body), papilledema, ascites, and skin changes including nail clubbing and hyperpigmentation [28, 29]. Neuropathy is the main feature and often precedes the diagnosis of osteosclerotic myeloma. Positive sensory symptoms and slowly progressive, predominately distal weakness occurs [30]. If untreated, POEMS syndrome has a poor prognosis with a median survival of 12–33 months [31].

Neuropathies associated with lymphoma are heterogeneous with variable prognosis depending on the type. Demyelinating forms may have a more favorable prognosis [32]. Aggressive B cell lymphoma, usually associated with proximal infiltration suggested by isolated radiculopathy, has a poor prognosis. Axonal multiple mononeuropathies related to distal infiltration have better outcomes [33–35].

Waldenstrom macroglobulinemia (WM) can result in peripheral neuropathy in up to 47 % of patients. Most patients with WM-related neuropathy complain of sensory loss and unsteady gait. Less commonly there is a predominant motor neuropathy which may be associated with elevated titers of IgM antibodies targeting ganglioside GM1 [36].

## Testing strategy

A general and detailed neurological exam should be performed to characterize the neuropathic phenotype. Tests should include a hematology panel as well as serum and urine protein electrophoresis with immunofixation. Table 1 describes diagnostic tests, including urinalysis, imaging studies, electrodiagnostic tests, and biopsies.

**Table 1:** Diagnostic tests for PPN

Test	Description	Comments
Erythrocyte sedimentation rate (ESR)	Venous blood sample.	ESR elevation is primarily due to increased levels of Ig (clonal or polyclonal) or fibrinogen
Serum total protein and albumin	Venous blood sample.	Protein elevated in:
	Normal range:	Monoclonal gammopathy
	Serum total protein: 6 to 8 g/dL	Dehydration
	Serum albumin: 4 to 6 g/dL	Myeloma
		Waldenstrom macroglobulinemia
		Sarcoidosis
		Collagen vascular disease
Serum protein electrophoresis	Venous blood sample.	Indicated if serum protein and/or globulin is elevated or clinical findings raise suspicion of monoclonal gammopathy.
		M protein, if present, is a discrete spike in the γ, β or α2 region.
		MGUS, M peak: 0.5 to 3 g/dL, amount directly related to probability of progression to multiple myeloma or a related plasma cell malignancy.
		Serum M spike present in 80 % of patients with myeloma.
Immunofixation	Venous blood sample.	Indicated when an M spike is found on serum electrophoresis or when clinical findings present suspicion of multiple myeloma, other plasma cell malignancy, amyloidosis, or WM.
	Monoclonal immunoglobulin:	
	MGUS: IgG (73 %), IgA (11 %), IgM (14 %), IgD, κ or λ light chains	Defines the heavy and light chain type of the abnormal serum protein, which can discriminate between MGUS, multiple myeloma, other plasma cell malignancies, WM, and amyloidosis.
	Multiple myeloma: IgG (50 %), IgA (20 %), IgD (few), free light chains (17 %)	
	WM: IgM-κ	
	Amyloidosis: IgG, IgA, IgD, IgM, κ or λ light chains; 30 % non-secretory	
	Osteosclerotic myeloma: IgG-λ or IgA-λ	
	Heavy-chain disease: IgG, IgA, IgM, no light chain	
Serum light chain quantitation	Venous blood sample.	Provides a rapid, accurate, quantitative measurement of λ and κ light-chain in serum.
	κ free light chain: 3.3 to 19.4 mg/L	Increased light chain levels are seen in most plasma cell disorders, especially the more malignant disorders such as multiple myeloma.
	λ free light chain: 5.7 to 26.3 mg/L	Free light chain (FLC) ratio may be a risk factor for progression to malignancy [66].
		Unlike urine Bence-Jones protein assays, results are not affected by changes in renal function.
		The test is expensive and not widely available.
Autoantibody panels	Normal:	Measures presence and titer of antibodies.
	Absence of antibody	Anti-MAG antibody assesses distal demyelinating sensory neuropathy.
		Anti-GM1 antibody assesses multifocal motor neuropathy.
		Anti-GQ1b antibody assesses Miller-Fisher.
		Please note that absolute absence of autoantibodies is not required for a “normal” test for many antibodies at different labs.
Cryoglobulins	Normal:	Serum blood specimen collected and separated while warm for cryoprecipitation over a period of up to 7 days.
	Less than 80 μg/ml	At very high cryoglobulin titer states, cryoprecipitates during blood collection produce structures on peripheral blood smears that may be mistaken for leukocytes or platelets by automated cell differential analyzers.
24-h urine protein quantification and electrophoreses	Detects excretion of monoclonal immunoglobulin.	Dipstick test for proteinuria primarily detects albumin and often misses M protein.
	Normal:	
	Urinary protein excretion less than 150 mg/day.	Recommended for patients with serum M spike or clinically-based suspicion of monoclonal gammopathy.
	Small amount of Bence-Jones protein not uncommon	
Urine immunofixation	Characterizes urinary monoclonal immunoglobulin following test of 24-h urine and should be done if serum M spike is greater than 1.5 g/dL.	Indicated if multiple myeloma, WM, primary amyloidosis, or a related disorder is suspected, even if routine urinalysis is negative for protein, 24-h urine is within normal limits, or if no M spike is seen on electrophoresis of concentrated urine sample.
Electrodiagnostic (electro-myelogram and nerve conduction studies)	Determines whether symptoms are due to a muscle or nerve disorder by measuring conduction velocities and the presence or absence of conduction blocks.	Determines whether the polyneuropathy is axonal or demyelinating.
		Tests help to localize the anatomic site of a lesion that is causing pain, and determine the presence of active denervation.
Bone marrow aspiration and biopsy	A sample is taken usually from the posterior superior iliac crest region.	Required if a high M protein level is found to investigate the possibility of multiple myeloma or lymphoma.
	Normal result is age-appropriate cellularity and lineage distribution and < 10 % plasma cells.	May reveal clinically inapparent involvement.
		Requires local anesthesia and the assistance of an attendant.
		Risk of infection and bleeding.
Radiographic skeletal bone survey	Two dimensional radiographs of the entire skeleton.	Survey detects lytic and sclerotic lesions as well as fractures which may be pathologic.
		There is a relatively high radiation exposure.
Cerebrospinal fluid analysis	Investigate CIDP and leptomeningeal lymphomatous infiltration.	Elevated protein level is common in PPN.
		Infiltration of the CNS by Non-Hodgkin’s lymphoma will show clonal lymphocytes.
		Viral infection may result in increased CSF lymphocytes but will not be clonal.
		Autoantibodies can be tested within the CSF but the results may differ depending on the laboratory used. Absolute absence of autoantibodies is not required for a “normal” test for many antibodies at different labs.
Nerve biopsy	Biopsy of the superficial peroneal nerve is ideal so that a muscle biopsy of the peroneus brevis muscle may be done simultaneously; other choices include sural or superficial radial sensory nerves.	Identifies abnormal density of small and large axons and abnormal myelin sheaths.
		Reserved for cases in which it is difficult to identify whether the process is predominantly axon degeneration or demyelination, or for cases where there is patchy, asymmetric, or focal involvement.
	Evaluates suspected cases of infiltrative neoplasms, paraproteinemic vasculitis, or amyloidosis.	A negative nerve biopsy does not exclude amyloid neuropathy.
		Please note that as with serum and CSF autoantibody testing, results may differ depending on the laboratory used.
Muscle biopsy	See “Nerve biopsy” for best incision site.	The procedure helps distinguish between an atypical neurogenic disorder and a primary myopathic disorder.
Fat biopsy	A normal result is no amyloid protein.	The test is most often done when there is suspicion of amyloidosis.
Skin biopsy	Examines the degree of myelination of small fiber neurons.	Helps ascertain the presence or absence of small fiber neuropathy.
		Epidermal nerve twig analysis via skin biopsy is sometimes done if small fiber neuropathy is suspected.
Whole-body computerized tomography scan	The scan can detect lymphadenopathy, hepatosplenomegaly, and ascites.	Intravenous contrast is usually required for better visualization of lymphoid structures.
Positron emission tomography scan	Functional images assess metabolic activity within various structures, including lymph nodes and may detect nodal, extranodal, and bone marrow involvement by lymphoma.	PET scan can be used concurrently with non-contrast CT scan to combine functional and anatomic imaging.
		Acute inflammation and infection can also result in increased uptake safety profile of the procedure, even though it is less sensitive than biopsy.

For patients who are found to have a paraproteinemia, diagnostic testing leans towards investigations of possible hematologic neoplasms. If none are discovered, then other diseases should be considered, including primary (AL) amyloidosis and cryoglobulinemia [22, 24]. Monoclonal proteins should be characterized by protein electrophoresis and immunofixation of the serum and the urine. Detailed electrophysiology includes determination of DML (distal motor latency)/MNCV (motor nerve conduction velocity)/TLI (terminal latency index), assessment for CB (conduction block)/ATD (abnormal temporal dispersion). If results of the CBC and/or peripheral smear examination are abnormal, a bone marrow biopsy is recommended, in consult with a hematologist. Even if the results of the CBC and/or peripheral smear examination are normal, then a bone marrow biopsy is still considered and usually recommended based on consultation with a hematologist before the diagnosis of MGUS is made. A skeletal X-ray survey can detect the presence of lytic lesions.

Cerebrospinal fluid (CSF) analysis including cytology and neuroimaging studies (MR neurography) may exclude leptomeningeal infiltration, especially in the presence of lymphoma. Nerve and muscle biopsy are occasionally performed to exclude infiltrative neoplasms, paraproteinemic vasculitis, or AL. If vasculitis is suspected, biopsy of the sural nerve is indicated. Alternatively, biopsy of the superficial peroneal nerve and peroneus brevis muscle obtains both nerve and muscle with a single incision and may increase the yield of identifying vasculitic pathology by up to 10 %. In the work-up of possible AL, abdominal fat aspiration biopsy is preferred over nerve biopsy due to the more favorable safety profile of the former procedure, despite lower sensitivity. If small fiber neuropathy is suspected, epidermal nerve twig analysis via skin biopsy may be performed.

### VEGF testing for POEMS

In patients with M-protein, endocrine and skin changes, and demyelinating polyneuropathy, testing for elevated vascular endothelial growth factor (VEGF) may help diagnose POEMS. A retrospective study found that VEGF levels in patients with POEMS syndrome were markedly elevated compared with patients with other plasma cell dyscrasias (*P* < .001), peripheral neuropathy (*P* < .001), and connective tissue disease/vasculitis (*P* < .009). The best VEGF cut-off for POEMS diagnosis was 146 pg/mL; however, a cut-off of 200 pg/mL had a specificity of 95 % with a sensitivity of 68 % in support of a POEMS diagnosis (level of evidence: 3) [37].

## Paraproteinemia treatment strategy

Many of the treatments for PPN are chemotherapy agents that may have significant impact on lifestyle. Psychological support for both the patient and family is often needed. As lifestyle modifications generally recommended in clinical practice, patients are counseled to remain as active as possible, abstain from alcohol, and stop smoking, if applicable. Several specialties may collaborate on treatment plans, including hematology for chemotherapy, neurology for pain management, radiation oncology for radiotherapy of plasmacytomas in POEMS syndrome, surgery for removal of solitary plasmacytomas in POEMS syndrome, and physical/occupational/recreational/speech/rehabilitation therapy [17]. Table 2 lists treatments directed to paraproteinemia.

**Table 2:** Treatment for paraproteinemia

Treatment	Indication	Dose	Major contraindications
Intravenous immune globulin (IVIG)	First line treatment for CIDP associated with M protein (off-label use)	2 g/kg intravenously for 3–5 days, followed by maintenance dose of 1 g/kg every 3–4 weeks, followed by clinical reassessment	Hyperprolinemia
	First line treatment for multifocal motor neuropathy (off-label)		Hypersensitivity to albumin
	Alternative treatment for IgM/A/G-MGUS (after rituximab, off-label use)		Immunoglobulin A deficiency
	Treatment of CANOMAD (off label use)		
Corticosteroids	First line treatment for CIDP associated with M protein (off-label use)	Prednisone:	Corticosteroid hypersensitivity
	An alternative treatment for IgM/A/G-MGUS (after rituximab or plasma exchange, off label use)	1 to 1.5 mg/kg/day orally	Fungal infection
		Several treatment dosing regimens are available and duration is not agreed upon.	
Azathioprine	Alternative immunosuppressive treatment (after IVIG or corticosteroids) for CIDP associated with M protein (off-label use)		Pregnancy (category D)
			Hypersensitivity to azathioprine
Rituximab	First line treatment for IgM-MGUS (off-label use)	Four weekly infusions of 375 mg/m2 rituximab [51]	Hypersensitivity to rituximab
	Alternative therapy for multifocal motor neuropathy (after IVIG, off label use)		
Chlorambucil	Alternative therapy for IgM-MGUS (off label use)	0.1 to 0.2 mg/kg orally daily for 3 to 6 weeks	Hypersensitivity to chlorambucil
			FDA pregnancy category D
Fludarabine	Alternative therapy for IgM-MGUS (off label use)	40 mg/m^2^ for 5 days every 28 days	Hypersensitivity to fludarabine
			FDA pregnancy risk category D
Melphalan	Treatment for POEMS	For POEMS (high dose): 10 mg/m^2^ for 4 days every 28 days [58]	Hypersensitivity to melphalan
	In combination with prednisone as treatment for AL	For AL: 0.15 mg/kg once daily for 7 days every 6 weeks, increasing the dose by 2 mg in each 6 week course. Should be used in combination with prednisone.	Should not be used in patients whose disease has demonstrated prior melphalan resistance
Plasmapheresis (plasma exchange)	First line treatment of IgG/A MGUS	3 to 5 exchanges every other day	Risks associated with frequent vascular access
	May be helpful in severe cases of cryoglobulinemia.		Risk of transmission of infective agents if fresh plasma is used as replacement fluid
			Anaphylaxis and allergic reactions may occur with reinfusion of plasma substitute
Autologous peripheral stem cell transplant	First line treatment of POEMS syndrome	Myeloablative doses of chemotherapy and/or radiation therapy followed by infusion of peripheral blood stem cells	Patients who undergo HCT are at risk for bacterial, viral, and fungal infections
	Alternative treatment of AL, used in combination with melphalan		Early adverse effects include: nausea, vomiting, diarrhea, mouth sores
			Later adverse effects include: cataracts, sterility, increased risk of other neoplasias
Radiation therapy	First line treatment of dominant sclerotic plasmacytoma in POEMS syndrome	Radiation therapy delivered to osteosclerotic lesions in doses of 40–50 Gy	Adverse effects depend upon the location of the area irradiated
			Increased risk of developing second malignancies in patients with Hodgkin’s Lymphoma

The mode and method of monitoring depends on the particular condition. Treatment decisions from a neurology standpoint usually are not based only on M-protein levels but rather the clinical picture, including severity, progression, and topography of motor deficit all which are important in selecting appropriate treatment. Additionally for MGUS patients, periodic (every 6–12 months) immunoglobulin quantitation and serum immunofixation may be done to determine changes in monoclonal protein level [8]. We consider treatment (always in consult with a hematologist) when the serum monoclonal protein rises above a concentration of 1.5 g/dL [38, 39]. Occasionally, serial electrodiagnostic studies are used to monitor disease response or progression [36]. Complications from the neuropathy itself include neuropathic ulcers and pain, Charcot joints, orthostasis, and predisposition to peripheral injury given lack of sensation [40].

PPN treatment first aims for paraprotein quantity reduction and the diminishing of cells producing the paraproteins, which may lead to improvement of neurological symptoms. IgM PPN sometimes responds to immunotherapies, but the potential benefits should be balanced against possible side effects, as well as the typically slow disease progression [18]. IgG and IgA PDN may be indistinguishable from chronic inflammatory demyelinating polyradiculoneuropathy clinically, electrophysiologically, and in response to treatment. The presence of paraprotein-related vasculitis or AL may also require treatment modification [19].

### Intravenous immune globulin (IVIG)

A systematic Cochrane review identified seven randomized controlled trials that examined the efficacy of any form of immunotherapy in reducing disability and impairment resulting from IgM anti-MAG paraprotein-associated demyelinating peripheral neuropathy. The primary outcome measure was the change in Neuropathy Impairment scale or Modified Rankin after six months, and secondary outcomes included shorter-term changes in impairment scale scores as well as paraprotein levels after six months. None of the seven trials provided adequate evidence to support immunotherapies based on the primary outcome measured at six months [41]. However, two short-term trials of IVIG showed statistically significant improvements in Modified Rankin Scale at two weeks and 10-m walk time at four weeks (level of evidence, 2). Other studies have indicated that IVIG was effective in 15 % to 20 % of patients [42–44]. A prospective study of 22 patients indicated that IVIG induced a short-term benefit in 50 % of patients with IgM paraprotein-associated neuropathies (11 of 19 patients had elevated anti-MAG antibodies) [45]. These findings suggest IVIG may provide benefit for some patients.

IVIG adverse effects may include allergic reactions, cephalgia, aseptic meningitis, hemolysis, renal adverse effects mostly in sucrose-based preparations, and hypercoagulable states (e.g., DVT, PE, and MI).

### Plasmapheresis (plasma exchange)

This blood purification procedure removes antibodies, thereby preventing them from binding their targets. The procedure removes the blood, separates blood cells from plasma, and returns purified blood, diluted with a plasma substitute, to the circulation [46]. Plasmapheresis is used for certain patients with IgG/A MGUS-associated neuropathy and may be helpful in severe cases of cryoglobulinemia. Plasmapheresis has only short term efficacy and must be repeated to maintain effectiveness. The procedure has limited utility for treatment of IgM-associated PPN.

A systematic review of treatments for IgG or IgA paraproteinemic peripheral neuropathy identified one relevant randomized controlled trial with 18 participants. Results showed plasma exchange had modest improvement over sham plasma exchange over a short-term follow up (level of evidence: 2) [47].

### Corticosteroids

A review of case reports and uncontrolled studies indicated that corticosteroids, when given in conjunction with other therapies, produced a response in about half of the patients with high anti-MAG IgM, but was seldom effective as a single therapy [43]. Corticosteroids have potential adverse effects on numerous organ systems (dermatologic, metabolic, cardiovascular, immune, gastrointestinal, central nervous system, bone), and patients receiving long-term or high doses of corticosteroids should be monitored for the development or worsening of these conditions. Long-term use (usually >3 weeks) or doses greater than physiological amounts (7.5 mg prednisone), may lead to clinically relevant suppression of the pituitary-adrenal axis, or exogenous Cushing syndrome. Corticosteroids may cause immunosuppression, which may mask signs of infection and increase patient susceptibility to infection. Patients should not receive live attenuated vaccines during therapy [48, 49].

### Azathioprine

After IVIG or corticosteroids, azathioprine is an alternative immunosuppressive treatment for CIDP [21]. The US FDA requires a boxed warning to advise of increased risk of neoplasia, particularly lymphoma (hepatosplenic T-cell lymphoma) and skin cancers associated with use of azathioprine. The drug is a purine analog that causes immune suppression and subnormal response to infections or vaccines. Infections should be treated vigorously and live vaccines avoided. There is risk of irreversible or delayed bone marrow suppression. Individuals with thiopurine methyltransferase (TPMT) deficiency may be unusually susceptible to myelosuppression. There is also a risk of pancreatitis and hepatotoxicity, which may be dose-related. A gastrointestinal hypersensitivity reaction characterized by severe nausea and vomiting has also been reported and may occur in the first few weeks of treatment. Hepatic or renal impairment may require a dose reduction. CBC, renal and liver function should be monitored periodically.

### Rituximab

This monoclonal antibody targets the CD20 antigen on B lymphocytes and is a first-line treatment for IgM-MGUS (off-label use). A small randomized study determined the effectiveness of rituximab versus placebo on symptoms of neuropathy in patients with anti-MAG IgM demyelinating polyneuropathy. Twenty-six patients were randomized to four weekly infusions of 375 mg/m^2^ rituximab or placebo. After 8 months, 4 of 12 rituximab-treated patients improved by at least one INCAT score (a measure of leg disability) compared with 0 of 13 patients taking placebo (*P* = .036) (level of evidence, 2) [50]. Another double blind placebo controlled trial of rituximab randomized 54 patients with IgM anti-MAG demyelinating neuropathy to receive rituximab or placebo. The primary outcome was the mean change in INCAT sensory score at 12 months, which showed no significant difference between treatment arms. However, with six patients in the rituximab group dropping out of the study, the per protocol analysis showed significant improvement in the rituximab group for secondary endpoints of INCAT disability scale and self-evaluation scale (*P* = .027 and *P* = .016, respectively) [51]. Both trials indicate statistically significant improvement in clinical rating scales (as secondary outcomes in some cases) suggesting that the use of rituximab for patients with anti-MAG IgM demyelinating polyneuropathy may provide benefit. Although some may not improve and the worsening is part of the disease progression, some patients may worsen after rituximab, especially if IgM levels are very high, similarly as with Waldenstrom’s macroglobulinemia [52–54].

### Chlorambucil

The alkylating agent chlorambucil is well tolerated by most patients but immunosuppression and bone marrow suppression may develop, which may lead to an increased susceptibility to infections or bleeding. Neutropenia can occur after the third week of chlorambucil therapy and continue for up to 10 days after the last dose. CBC should be monitored weekly.

### Fludarabine

Fludarabine is used as alternative therapy for IgM-MGUS, and the efficacy of the cytotoxic purine antimetabolite has been demonstrated in very small case series, both alone and in combination with rituximab [55, 56]. Use of fludarabine can cause severe bone marrow suppression. Life-threatening and sometimes fatal autoimmune hemolytic anemia and immune thrombocytopenic purpura have been reported following one or more cycles of fludarabine therapy in approximately 5 % of patients. Nausea/vomiting during intravenous fludarabine treatment is common. CBC should be monitored weekly.

### Melphalan

Melphalan in combination with prednisone may reduce the monoclonal protein level and even prolong survival in AL, but the specific effect of this regimen on peripheral neuropathy is not known [57]. Melphalan is used for treatment of POEMS syndrome. A study from China on 31 patients with POEMS reported that high-dose melphalan plus dexamethasone resulted in good hematologic and neurologic responses [58]. The drug may cause a secondary malignancy; melphalan is leukemogenic in humans. Myelosuppressive effects of melphalan can increase the risk of infection or bleeding. The dosage of melphalan should be reduced or therapy discontinued at the first signs of neutropenia or thrombocytopenia. CBC should be monitored closely.

## Symptomatic treatment strategy

Symptomatic treatment of the neuropathy itself usually involves membrane stabilizers, tricyclic anti-depressants, and/or serotonin-norepinephrine reuptake inhibitors. Guidelines from the American Academy of Neurology summarize evidence-based information on pharmacologic and nonpharmacologic treatments for painful neuropathy [59].

### Gabapentin

A structural analogue of the inhibitory neurotransmitter γ-aminobutyric acid (GABA), gabapentin is a first line treatment for neuropathic pain and is effective for a variety of neuropathic pain conditions. It exerts its anti-nociceptive effect by binding the alpha-2 delta calcium channel in the dorsal horn of the spinal cord. It should be used with caution in renal insufficiency; the dose must be adjusted.

### Pregabalin

Designed as a more potent successor to gabapentin, pregabalin is effective for a variety of neuropathic conditions. It should be used with caution in renal insufficiency; the dose must be adjusted.

### Valproate

Valproate agent should be used with caution in hepatic impairment; dose reduction is required. The US FDA requires a boxed warning to advise of hepatic failure resulting in fatalities and to advise of teratogenic effects, such as neural tube defects, when used in pregnancy.

### Dextromethorphan

N-methyl-d-aspartate (NMDA) antagonists such as dextromethorphan block the activation of NMDA receptors, which is contributory to development of central sensitization resulting in hyperalgesia, hyperpathia, allodynia, and reduced functionality of opioid receptors. Side effects may include light-headedness, drowsiness, visual disturbances, and hot flushes; the agent should be used with caution in patients who are sedated or debilitated [60].

### Tramadol

The mixed opioid, mu agonist and inhibitor of uptake of serotonin and norepinephrine, should be used with caution in renal and hepatic impairment; dose reduction is required. In rare instances anaphylactic reactions have been reported. The agent may cause CNS depression, which may impair physical or mental abilities.

### Duloxetine

Duloxetine is a serotonin-norepinephrine reuptake inhibitor (SNRI) used for symptomatic treatment for peripheral neuropathy. It is FDA approved for the treatment of diabetic peripheral neuropathic pain (DPNP). The US FDA requires a boxed warning to advise of suicidal thinking and behavior in children, adolescents, and young adults (18 to 24 years of age) with major depressive disorder (MDD) and other psychiatric disorders.

### Amitriptyline

Amitriptyline is the most widely used tricyclic antidepressant (TCA), and is used off-label for symptomatic treatment of peripheral neuropathy. The US FDA requires a boxed warning to advise of suicidal thinking and behavior in children, adolescents, and young adults (18 to 24 years of age) with major depressive disorder and other psychiatric disorders.

### Venlafaxine

The serotonin/norepinephrine reuptake inhibitor (SNRI) venlafaxine is used off-label for symptomatic treatment of peripheral neuropathy. The US FDA requires a boxed warning to advise of suicidal thinking and behavior in children, adolescents, and young adults (18 to 24 years of age) with major depressive disorder and other psychiatric disorders. In patients with renal impairment (GFR 10–70 ml/min), reduce total daily dose by 25 to 50 %. In patients with mild to moderate hepatic impairment, reduce total daily dose by 50 %.

### Autologous peripheral stem cell transplantation

As a first line treatment for POEMS syndrome and alternative treatment (in combination with melphalan) for AL, autologous peripheral stem cell transplantation entails administration of myeloablative doses of chemotherapy and/or radiation therapy followed by infusion of peripheral blood stem cells [61, 62].

Granulocyte colony stimulating factor (G-CSF) is given to stimulate peripheral blood stem cells followed by hematopoietic stem cell mobilization four to six days later. Peripheral blood progenitor cells (PBPCs) are mobilized using a variety of techniques. Following initiation of a mobilization regimen, patients are monitored by peripheral blood CD34 counts. Apheresis begins when the peripheral CD34+ counts have reached a target level (i.e., 10 CD34 cells/μl). After completion of the preparative chemotherapy, PBPCs are reinfused. A period of pancytopenia follows and red blood cell and platelet transfusions are administered as necessary while G-CSF is used to speed neutrophil engraftment. Lifelong follow up is necessary to monitor for complications and recurrence. Patients are at risk for bacterial, viral, and fungal infections. Early adverse effects include nausea, vomiting, diarrhea, and mouth sores; later adverse effects include cataracts, sterility, and increased risk of other neoplasias.

Efficacy of the procedure for peripheral neuropathy symptom improvement is based on small case series, and data suggest that most patients achieve at least some neurologic improvement. A small study of 9 patients with POEMS syndrome evaluated the extent and time course of neurologic improvement after autologous peripheral blood stem cell transplantation. Within 3 months, neurologic improvement began, and all the patients showed substantial neurologic recovery during the next 3 months. At the end of follow-up periods (8 to 49 months, median 20 months), neuropathy was still improving and no patients had recurrence of symptoms (level of evidence: 3) [62].

### Radiation therapy

Radiation therapy as a first line treatment for dominant sclerotic plasmacytoma in POEMS syndrome is delivered to osteosclerotic lesions in doses of 40–50 Gy. More than 50 % of patients treated with radiation show improvement of the neuropathy, but improvement in some patients may be delayed, occurring after six months or longer [31].

### Physical, occupational, speech, recreational, and rehabilitative therapies

For training in performance of activities of daily living, physical therapy that focuses on compensatory strategies to accommodate for limbs with a loss of sensation and weakness is often done by patients with peripheral neuropathies. Amyloidosis as a paraprotein can cause gastroparesis/dysphagia, which is also seen in CANOMAD and can result from medication toxicity. Speech therapy focused on training in swallowing may attenuate symptoms in patients suffering gastroparesis or dysphagia [63, 64]. Recreational therapy helps patients recover basic motor functioning, to build confidence and socialize more effectively. Treatments may incorporate arts and crafts, sports, games, dance, drama, and/or music. Patients with chronic disease, especially the elderly, who are isolated and at risk for depression, may benefit from these therapies, which can improve socialization and diminish isolation.

### Orthotics

For the prevention of foot ulcers and infections, orthotics are molded cushion inserts for the foot that distribute pressures, reduce high stress areas, and provide shock absorption.

### Acupuncture

A method of Chinese medicine aims to produce analgesia by insertion of sharp, thin needles into the body at very specific points. Acupuncture has produced improvement in both subjective symptoms and objective nerve conduction study findings [65].

### Transcutaneous electrical nerve stimulation (TENS)

TENS is a form of electroanalgesia that reduces pain through nociceptive inhibition at the presynaptic level in the dorsal horn of the spinal cord. A TENS unit consists of 1 or more electrical-signal generators, a battery, and a set of electrodes. The TENS unit is small and programmable, and the generators can deliver trains of stimuli with variable current strengths, pulse rates, and pulse widths. Patients may experience skin irritation due to drying out of the electrode gel. TENS is contraindicated in patients with a demand-type pacemaker.

## Conclusions

Individual paraproteinemic disorders exhibit distinct neuropathic phenotypes, often with characteristic, identifiable clinical features. Frequently, prognoses are not well-defined, and in many cases, unknown. Testing should include a general and detailed neurological exam to characterize phenotype, and may include various diagnostic tests depending on the case. PPN treatment often calls for collaboration among several specialties, including hematology, neurology, radiation oncology, surgery, and physical/occupational/recreational/speech/rehabilitation therapy. Treatments for PPN often involve chemotherapy agents that may significantly impact a patient’s lifestyle. For these individuals and their families, psychological support is often needed.
